# A Comparative Evaluation of the Erosion Level of Commonly Prescribed Pediatric Liquid Medicines on Primary Teeth: An In-Vitro Surface Profilometry and Scanning Electron Microscopic Study

**DOI:** 10.7759/cureus.80370

**Published:** 2025-03-10

**Authors:** Shanthosh Raj Srinivasan, Anjana M, Vignesh K C, Ramadevi R P, Poornima V, Deebiga K

**Affiliations:** 1 Dentistry, Employees' State Insurance Corporation (ESIC) Medical College and Hospital, Chennai, IND; 2 Pediatric Dentistry, Ragas Dental College and Hospital, Chennai, IND; 3 Pediatric and Preventive Dentistry, Sri Ramachandra Dental College and Hospital, Sri Ramachandra Institute of Higher Education and Research (Deemed to be University), Chennai, IND; 4 Pediatric and Preventive Dentistry, Madha Dental College and Hospital, Chennai, IND; 5 Pedodontics and Preventive Dentistry, Ragas Dental College and Hospital, Chennai, IND

**Keywords:** enamel erosion, liquid medications, oral health, pediatric dentistry, surface properties

## Abstract

Introduction

Maintaining good oral health is crucial for the overall health, growth, and development of children. Dental caries is the most widespread oral disease and the most common infectious condition affecting the mouth. Epidemiological studies have shown a frequent occurrence of enamel erosion in young people. Pediatric liquid analgesics (PLAs) are frequently prescribed and well-accepted by young patients. However, prolonged use of these medications can increase the risk of dental caries and dental erosion. This study aims to evaluate the erosive potential of commonly prescribed pediatric liquid medicinal syrups on primary teeth enamel. The objectives include assessing changes in the enamel prismatic pattern of deciduous teeth using scanning electron microscopic (SEM) analysis and comparing the erosive effects of these medications through surface profilometric analysis.

Methodology

The current study model was designed to stimulate clinical erosion of tooth surfaces when exposed to frequently prescribed medications. Test samples - a convenient sampling of 40 caries-free extracted primary incisors - were used for the study. The commonly prescribed pediatric medications were selected: (a) Ibugesic plus syrup, (b) Novomox syrup, (c) Dexorange syrup, (d) Benadryl syrup, and (e) Asthalin syrup. The agitation of pediatric liquid medicaments (PLMs) was done for about one minute. The teeth samples were changed accordingly, from artificial saliva to respective PLM, and agitated for one minute. Teeth samples in PLMs were processed by changing them periodically, thrice daily, for five consecutive days. PLMs were changed every day; the air-tight containers were washed and wiped with sterile cotton. Processed samples were sent for surface profilometry analysis (27 teeth) and SEM study (9 teeth). The results from the profilometric analysis and SEM analysis were tabulated, and data were analyzed using the software SPSS.

Results

The results showed marked changes in the levels of erosion between the control and test groups. It shows that there was a significant difference between the control and other test groups (p-value: 0.028). Different levels of erosion were seen on the tooth samples with different medications. This study reveals that these combinations of drugs have a high erosive potential, which shows increased cariogenicity.

Conclusion

Based on the experimental conditions of this study, it can be concluded that acidic medications contribute to enamel erosion. The intake of medicinal syrups by children is often necessary, and their harmful effects on oral and dental structures are unavoidable.

## Introduction

Maintaining oral health is vital for the overall well-being, growth, and development of children. Dental caries, the most frequent oral disease, also stands as the most common infectious disease affecting the mouth. The predominant theory of dental caries suggests that acids produced by bacterial fermentation of dietary carbohydrates (sugars) are the primary cause of dental caries [1]. However, certain diseases or medications can heighten the risk or severity of caries. Dental erosion is described as the "irreversible loss of tooth structure due to chemical dissolution by acids, without bacterial involvement" [2]. Acid sources can be intrinsic, such as acid reflux, vomiting, or regurgitation from gastrointestinal conditions, or extrinsic, originating from acidic foods and beverages [3].

In recent decades, epidemiological studies have shown a widespread occurrence of enamel erosion in youth, leading to a considerable focus on the mechanisms behind dental erosion [4]. Many liquid medications, frequently used by children with chronic conditions, contain high levels of sugar and pose an increased risk for dental caries. Liquid forms of medicine are popular for pediatric use due to their ease of administration [5]. However, some inactive ingredients in these medications can harm dental tissues because of their low pH. Certain medications include acidic components to maintain chemical stability and control tonicity [6].

Syrups with a pleasant taste have been long utilized in pediatric medicine to encourage adherence to treatments. Chronically ill young children often take various oral liquid medications for health maintenance or improvement. Pharmaceutical companies enhance the taste of these liquid medications with sucrose to increase palatability, ensure compliance, and act as a preservative [7]. Moreover, these syrups include ingredients that enhance appearance, bioavailability, and stability. These inactive agents, however, pose risks, such as dental caries and erosion. Pediatric liquid analgesics (PLAs) are commonly prescribed and well-received. Nevertheless, prolonged use of these medications can increase the risk of dental caries and erosion [8].

Pediatric liquid medicaments (PLMs) are notably more palatable for children compared to their tablet counterparts, primarily due to their sweetened formulations. Sucrose is a common additive in the pharmaceutical industry. However, the frequent intake of sugars can be swiftly fermented by oral bacteria, generating enough acid to erode dental enamel [9]. Acids are routinely incorporated into medications as buffering agents to maintain chemical stability, control tonicity, or ensure physiological compatibility. Additionally, acids enhance flavor and facilitate acid-base reactions that disperse effervescent and dispersible tablets upon contact with water. When the pH of these medications falls below 5.5, they can trigger dental erosion. While the active ingredients in these medicines are essential for health maintenance or improvement, certain inactive components can lead to dental caries and erosion [10]. Given this context, this study aims to assess the erosive potential of commonly prescribed pediatric liquid medicinal syrups on the enamel of primary teeth. The objectives are to determine the variations in the topographic enamel prismatic pattern of deciduous teeth upon exposure to various commonly prescribed pediatric medications using scanning electron microscopic (SEM) analysis and to compare the erosive effects of these medications through surface profilometric analysis.

## Materials and methods

Study design and setting

**Figure 1:**
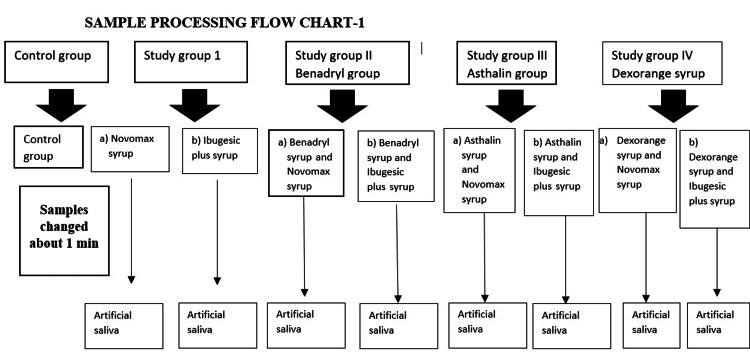
Sampling process

The current study was designed as an in vitro experimental study to simulate the clinical erosion of tooth surfaces when exposed to frequently prescribed medications. A convenient sampling of 40 extracted, caries-free primary incisors was used, with the teeth stored in distilled water prior to processing. The Ethical Committee of Dr. M.G.R. Educational and Research Institute, Chennai, India, obtained permission for the study (approval no. DRMGRDU/TMDCH/2015-16/0205020). The specimens analyzed were obtained from the Department of Pediatric Dentistry and Preventive Care at Thai Moogambigai Dental College and Hospital, Chennai, India. The principal investigator requested and acquired permission from the head of the department for sample collection. Samples were collected by placing a bottle with demineralized water in undergraduate and postgraduate clinics, and all samples were stored in demineralized water at room temperature until used for the study. The sampling process is explained in Figure 1.

Selection criteria

The inclusion criteria for the study were extracted primary maxillary incisors demonstrating exfoliating mobility. The exclusion criteria included decayed teeth, teeth with evidence of fracture, and teeth with discoloration.

Data sources and variables

Before mounting the teeth, they were cleaned with pumice slurry, and the roots were cut along the cemento-enamel junction. An acrylic plug was kept at the base of the tooth, and they were stored in demineralized water at room temperature. Artificial saliva was selected according to the study by Ruissen et al. [11]. The commonly prescribed pediatric medications were selected according to Kulkarni et al. [12], including Ibugesic plus syrup, Novomox syrup, Dexorange syrup, Benadryl syrup, and Asthalin syrup. These medications were distributed into groups for testing, as shown in Table 1: control group, Novomax syrup (group Ia), Ibugesic syrup (group Ib), combination of Benadryl plus Novomax (group IIa), Benadryl plus Novomax (group IIb), Asthalin plus Novomox (group IIIa), Asthalin plus Ibugesic syrup (group IIIb), Dexorange plus Novomax (group IVa), and Dexorange plus Ibugesic (group IVb).

**Table 1:** Distribution of samples

Groups	No. of samples	
Control group	04	Control group (artificial saliva)
Ia	04	Novomox syrup
Ib	04	Ibugesic plus syrup
IIa	04	Benadryl syrup and Novomax syrup
IIb	04	Benadryl syrup and Ibugesic plus syrup
IIIa	04	Asthalin syrup and Novomax syrup
IIIb	04	Asthalin syrup and Ibugesic plus syrup
IVa	04	Dexorange syrup and Novomax syrup
IVb	04	Dexorange syrup and Ibugesic plus syrup

The synthetic saliva was formulated following the method described by Ruissen et al. [11], utilizing three commercially available saliva substitutes: Xialine 1, Xialine 2, and Saliveze. Xialine 1 and Xialine 2 are composed of xanthan gum, whereas Saliveze is based on carboxymethylcellulose. According to Tupalli et al. [13], a total of 40 teeth were immersed in artificial saliva and then transferred into their respective containers for processing using sterile tweezers. Teeth were then transferred to group 1a, Novomox; group Ib, Ibugesic syrup; group IIa, Benadryl and Novomox; group IIb, Benadryl and Ibugesic syrup; group IIIa, Asthalin and Novomox; group IIIb, Asthalin and Ibugesic; group IVa, Dexorange and Novomox; and group IVb, Dexorange and Ibugesic. Continuous agitation was done for one minute in each 5 mL of solution using a stirrer, and a stopwatch was set for one minute for accuracy. The teeth samples were transferred back to artificial saliva, rinsed with distilled water, and the process was repeated thrice daily for about five days, following the American Association of Pediatric Dentistry (AAPD) guidelines for prescribing medications for children.

Teeth samples were periodically changed from artificial saliva to the respective PLM and agitated for one minute. They were processed by changing them thrice daily for five consecutive days, with PLM changed daily. The air-tight containers were washed and wiped with sterile cotton. Processed samples were sent for surface profilometry analysis (27 teeth) and SEM study (9 teeth) at the Sophisticated Analytical Instruments Facility (SAIF) and the Microelectronics and MEMS (Microelectromechanical Systems) Research Facility, within the Department of Electrical Engineering at IIT Madras.

Surface roughness, or surface texture, quantifies the finish of a surface by evaluating its topographical features. The SEM used was a JEOL JSM-5800 (JEOL Ltd., Tokyo, Japan), operating at 15 kV, and is highly effective for examining surface morphology. The data were analyzed using computer-processed micrographs with the Hewlett-Packard Ultra VGA 1600 device (HP, Palo Alto, CA, USA) and xT Microscope Server software. The erosion of primary teeth was classified according to Silverstone et al. [14] into five etching patterns: Type 1 etching pattern: the prism cores are predominantly eroded, leaving the surrounding prism peripheries undisturbed. Type 2 etching pattern: the prism cores are mostly preserved, while the surrounding prism peripheries undergo selective demineralization. Type 3 etching pattern: this pattern combines characteristics of both Type 1 and Type 2. Type 4 etching pattern: the surface exhibits noticeable pitting. Type 5 etching pattern: the result is a flat, smooth surface following etching.

Statistical analysis

The findings from the profilometric assessment were compiled and subjected to statistical analysis to ascertain the significance of the observed changes. The data were processed using IBM SPSS Statistics for Windows, Version 22 (Released 2013; IBM Corp., Armonk, NY, USA). Following the results of the normality test, non-parametric statistical techniques were employed. The Kruskal-Wallis test was utilized to compare surface profilometric values (µm) across different groups and syrups, with subsequent pairwise comparisons conducted using the Bonferroni adjustment and the Mann-Whitney test. For comparisons between groups, the Mann-Whitney test was used. The significance threshold was set at 5% (α = 0.05).

## Results

**Figure 2:**
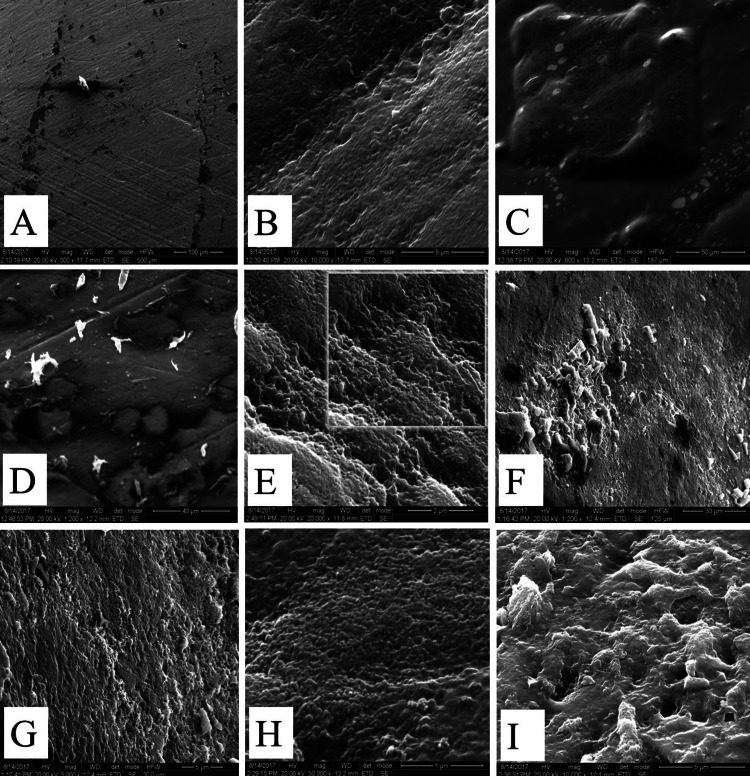
Characteristic comparison of surface morphology of control and test group samples after treated with medicaments Panel (A) depicts the control group. In this, the surface does not reveal any irregularities in enamel. Panel (B) depicts Novomox syrup (group Ia), which shows the fibrous appearance seen in the exposure of enamel. The surface demineralization was restrained to the exposure of enamel prisms in perikymata furrows due to Novomox syrup (type 1 pattern of Silverstone). Panel (C) depicts Ibugesic syrup (group Ib), which shows that the surface of enamel shows a fibrous appearance (type 2 pattern of Silverstone). Panel (D) depicts Benadryl and Novomox syrup (group IIa), which shows disturbance of collagenous fibers (etched prism pattern present). Panel (E) depicts Benadryl and Ibugesic syrup (group IIb), which shows loss of surface enamel exposure, with irregular rods (sporadic rod ends seen) (type 3 pattern of Silverstone). Panel (F) depicts Asthalin and Novomox (group IIIa), which shows the presence of crater formation due to exposure to Asthalin syrup (type 4 pattern of Silverstone). Panel (G) depicts Asthalin and Ibugesic (group IIIb), which shows visible enamel microcavities developed (type 4 pattern of Silverstone). Panel (H) depicts Dexorange and Novomox (group IVa), which shows opening of enamel rods, and porosity can be seen in the enamel surface (type 3 pattern of Silverstone). Panel (I) depicts Dexorange and Ibugesic (group IVb), which shows irregular erosion of cavities and premature elimination of the core segments of enamel prisms (type 3 pattern of Silverstone).

Figure 2 shows the characteristic comparison of surface morphology between control and test group samples after treatment with medicaments, as analyzed by SEM.

Table 2 shows the descriptive statistics for the surface profilometry. The results showed marked changes in the levels of erosion between the control and test groups.

**Table 2:** Descriptive values of analysis by surface profilometry

Group no.	Group	N	Mean	Std. dev	Median	1st quartile	3rd quartile
	Control	04	2.68	0.38	2.55	2.38	3.11
Ia	Novomox	04	3.07	0.21	3.08	2.85	3.27
Ib	Ibugesic	04	4.15	1.41	4.26	2.69	5.51
IIa	Benadryl + Novmox	04	3.35	0.20	3.30	3.17	3.57
IIb	Benadryl + Ibugesic	04	5.19	0.86	5.48	4.22	5.86
IIIa	Asthalin + Novomox	04	5.02	1.85	3.99	3.91	7.16
IIIb	Asthalin + Ibugesic	04	4.78	2.14	4.02	3.13	7.20
IVa	Dexorange + Novomox	04	6.58	2.62	5.91	4.36	9.47
IVb	Dexorange + Ibugesic	04	7.34	3.24	5.59	5.34	11.08

Table 3 shows the comparison of surface profilometric values between groups using the Kruskal-Wallis test. A p-value less than 0.05 is considered statistically significant. It shows that there was a significant difference between the control and other test groups (p-value: 0.028). It shows that there was a different level of erosion seen on the tooth samples with different medications.

**Table 3:** Comparison of surface profilometric values between groups *A p-value less than 0.05 is considered statistically significant, and the table shows the comparison of surface profilometric values between groups using the Kruskal-Wallis test.

Group no.	Group	N	Mean rank	Chi-square	p-value
	Control	04	3.00	17.23	0.028*
Ia	Novomox	04	6.00		
Ib	Ibugesic	04	13.00		
IIa	Ben+Novmox	04	9.67		
IIb	Ben+Ibugesic	04	18.67		
IIIa	Ast+Novomox	04	16.33		
IIIb	Ast+Ibugesic	04	15.33		
IVa	Dex+Novomox	04	22.00		
IVb	Dex+Ibugesic	04	22.00		

Table 4 shows the intergroup comparison using the Bonferroni-adjusted Mann-Whitney test. A p-value less than 0.05 is considered statistically significant. The results show a statistically significant difference between Control vs. Dexorange and Novomox groups (p-value: 0.039) and Control vs. Dexorange and Ibugesic groups (p-value: 0.041), revealing that these combinations of drugs have a high erosive potential, which shows increased cariogenicity.

**Table 4:** Intergroup comparison of profilometric values *A p-value less than 0.05 is considered statistically significant, and the table shows the intergroup comparison using the Bonferroni-adjusted Mann-Whitney test.

Group no.	Group	p-value
Ia	Control vs. Novomax	0.999
Ib	Control vs. Ibugesic	0.999
IIa	Control vs. Benadryl + Novmox	0.999
IIb	Control vs. Benadryl + Ibugesic	0.563
IIIa	Control vs. Asthalin + Novomox	0.999
IIIb	Control vs. Asthalin + Ibugesic	0.999
IVa	Control vs. Dexorange + Novomox	0.039*
IVb	Control vs. Dexorange + Ibugesic	0.041*

## Discussion

Primary teeth are more susceptible to erosion due to their lower mineral content compared to permanent teeth, and their enamel is less developed. Children who regularly use liquid medications are at risk, as some inactive components in these drugs can contribute to dental caries and erosion. Research has shown that certain PLMs are both acidogenic and cariogenic in nature.

The methodology and materials, including sample sizes and types of commonly used liquid medicines, were based on the study by Tupalli et al. [13]. The integrated examination of the chemical characteristics of acidic compounds and their effects on tooth erosion has been extensively applied. It is recommended that enamel demineralization occurs when the surface pH drops below 5.5, and numerous PLMs possess a pH level that could be detrimental to enamel. Laboratory studies have shown that these medications can reduce enamel hardness, increase roughness, alter its structure, and promote calcium loss. This study aimed to compare the effects of various pediatric drugs on enamel hardness and morphology, based on exposure time.

Feigal [15] provided evidence showing that continuous use of sucrose-based medicines led to dental caries and gingivitis. Further studies by Greenwood et al. [16] confirmed the cariogenic and acidogenic nature of these preparations. The erosive potential of acids in foods and medications is influenced by (1) the amount of acid present, (2) the concentration of hydrogen ions (pH), and (3) the strength of the acid, or its tendency to release hydrogen ions (pKa).

The effects of these PLMs on primary enamel were analyzed using SEM. An etching pattern was observed on nearly all enamel samples. Most samples exhibited large, crater-like structures, with variability in size and depth across specimens. The greatest surface changes were noted in the group receiving Dexorange and Ibugesic plus syrup (group IVb). The enamel prism pattern observed was consistent with a type III etching pattern, where the prism core material was selectively removed, leaving a honeycomb-like appearance, similar to findings by Babu et al. [10].

In the current study, SEM images revealed that the enamel surface was mostly smooth, with slight etching, showing faint scale outlines. The surface topography resembled the type III etching pattern, typically linked with aprismatic enamel. Previous research by Kiran et al. [17], Cavalcanti et al. [18], Passos et al. [19], and Blevins [20] has shown that PLMs with low pH levels can initiate dental demineralization and cause surface erosion by directly affecting the enamel.

Limitation of the study

Given that the erosion observed in this study was induced under controlled laboratory conditions, the findings may not be fully applicable to real-world in vivo scenarios.

## Conclusions

Based on the experimental conditions in this study, it can be inferred that acidic medications contribute to enamel erosion. The use of medicinal syrups in children is often necessary, but their negative impact on oral health is significant. To mitigate these effects, it is advisable to rinse the mouth with water immediately after syrup ingestion, incorporate calcium, fluoride, or phosphate into the formulations, and administer the medication during mealtimes. Caregivers should be encouraged to monitor or assist young children to ensure proper oral hygiene. Additionally, using artificial sweeteners instead of sugar in medications can help prevent the harmful effects associated with sugar.
